# The effects of serum TC, HDL, APOA-I levels on the prognosis of sepsis patients

**DOI:** 10.1186/2197-425X-3-S1-A874

**Published:** 2015-10-01

**Authors:** T Zhang, L Tong, CJ Cai

**Affiliations:** Guangzhou First People's Hospital, Guangzhou, China; The 1st Affiliated Hospital, Sun Yat-Sen University, Guangzhou, China

## Introduction

Cholesterol (total cholesterol, TC) as the part of the fat plays an important role in maintaining the integrity of cell membrane and generating certain of steroids as well as fat-soluble vitamins. Therefore, low levels of cholesterol in patients with sepsis maybe have adverse effects [1, 2]. Some studies have showed that high-density lipoprotein (HDL), apolipoprotein A-I (apoA-I) seem to be associated with the risk of infection by neutralizing the lipopolysaccharide(LPS) which was the most critical initiation factor in septic shock [3, 4].

## Objectives

To observe the relationship between the levels of total cholesterol (TC), high-density lipoprotein (HDL), apolipoprotein A-I (apoA-I) and the severity of sepsis. To analysis these markers' predictive value for sepsis.

## Methods

This is an retrospective observational study. A total of 37 patients hospitalized in the surgery intensive care unit of the Third Affiliated Hospital of Sun Yat-Sen University from December 2013 to December 2014 were enrolled in the study. All patients were divided into two groups according to the status after 28 days. The correlations between the lipoprotein and inflammatory cytokines, the independent predictive factors for sepsis were analyzed. Receiver-operating-characteristic curve (ROC) was used to evaluate the cut-off of TC, HDL, apoA-1 and APACH II as well as their predictive value for 28days mortality.

## Results

There was negative correlation between HDL, TC, apoA-1 and tumor necrosis factor-α(TNF-a), interleukin (IL-6) separately. Whereas these lipoprotein indexes didn't have significant correlation with neutrophil. The cut-off point of TC, HDL, apoA-I, APACHEII score were 1.46 mmol/l, 0.175 mmol/l, 0.525 g/l, 20.5 points. apoA-I has the highest diagnostic and predictive value.

## Conclusions

The levels of TC, HDL, apoA-I can reflect the degree of inflammatory response to the sepsis and the severity of the disease. TC, HDL, apoA-I, APACHEII within 24 hours of sepsis have predictive value. But apoA-I was the highest diagnostic one.

## Grant Acknowledgment

Thanks Mrs Tong to collect the data, recruit the cases.
